# Targeted Sanger Sequencing of a Cluster of COVID-19 Cases in the Surgical ICU of a Non-COVID Hospital: Lessons Learned

**DOI:** 10.7759/cureus.44755

**Published:** 2023-09-05

**Authors:** Jyothi Embekkat Kaviyil, Kavita Raja, Rakhal Gaitonde, Sreekumar Easwaran, Kumari Kala V, Dinoop Korol Ponnambath

**Affiliations:** 1 Microbiology, Sree Chitra Tirunal Institute for Medical Sciences and Technology (SCTIMST), Trivandrum, IND; 2 Public Health, Sree Chitra Tirunal Institute for Medical Sciences and Technology (SCTIMST), Trivandrum, IND; 3 Virology, Institute for Advanced Virology, Trivandrum, IND; 4 Nursing, Sree Chitra Tirunal Institute for Medical Sciences and Technology (SCTIMST), Trivandrum, IND

**Keywords:** n501i, sars-cov-2, sanger sequencing, s gene sequencing, epidemiological investigation, covid-19

## Abstract

Small clusters of infection due to SARS-CoV-2 in a non-COVID-19 healthcare facility can disrupt services. Here, we investigated a cluster of SARS-CoV-2 cases by targeted Sanger sequencing and clinical epidemiological methods in a non-COVID-19 super-specialty hospital. Epidemiological data were collected in a blinded manner using a proforma to find the risk factors associated with infection. Targeted Sanger sequencing of the spike protein receptor binding domain (RBD) coding region was performed on all the available real-time reverse transcription polymerase chain reaction (RT-PCR)-positive samples that included a patient, his mother, and 11 healthcare workers (HCWs) to determine any genomic variations in the samples from the cluster. All positive cases were due to the Delta variant. However, it detected a unique mutation, N501I, in the RBD region of the SARS-CoV-2 strains. The viral genome extracted from the mother’s sample lacked the mutation, thus excluding her from the cluster and pointing out that the outbreak was nosocomial, leading to a focus on infection control measures. Though whole genome sequencing is more universally accepted, in this study, targeted sanger sequencing provided a rapid and cost-effective solution to correctly delineate between the actual cases that form the cluster and other community cases in a pandemic situation.

## Introduction

The COVID-19 pandemic has been a devastating global public health crisis that has led to substantial socioeconomic disruption, morbidity, and mortality. The world has had to continuously grapple with successive waves of the COVID-19 pandemic since December 2019, fueled by the emergence of various viral variants [1]. The setting of this study is the congenital heart surgery unit of a non-COVID-19 super specialty referral hospital. In this hospital, infection control protocols were reviewed and updated for airborne infections, patients were screened for SARS-CoV-2 before admission, and weekly surveillance of all staff was instituted. Despite precautions, small clusters of COVID-19 infection developed in the wards and ICUs, necessitating quick containment to avert a total shutdown. In the waning phase of the second wave (Delta variant in India), a cluster of COVID-19 cases occurred in the congenital heart surgery unit (CH unit) from October 15 to 27, 2021, which prompted the investigation.

The setting: On October 19, 2021, a post-operative 11-year-old patient developed fever and increased secretions while on a ventilator, needing increased suction and close nursing care. On October 21, 2021, his mother tested positive for SARS-CoV-2 by RT-PCR. Suspicion about the change in the condition of the patient arose following the positive test result of the mother, and the patient was tested on October 22, 2021. He tested positive for SARS-CoV-2 by RT-PCR. Meanwhile, two nurses attending to the patient became symptomatic and tested themselves at another laboratory. They reported to the infection control team that they were infected. This alerted the infection control team to the occurrence of a possible cluster. As per hospital policy, the infection control team tracked all the contacts and, on October 23, 2021 screened them for SARS-CoV-2 by RT-PCR, regardless of whether they were symptomatic or asymptomatic. On screening, in addition to the patient and the mother, 13 healthcare workers (HCWs) tested positive for SARS-CoV-2 by RT-PCR during the period from October 15 to 27, 2021, out of 72 tested (13/72). Only 11 positive samples from the 13 HCWs and the two samples from the patient and mother were available for analysis by tSS (n=13). Samples from two HCWs who tested themselves at another laboratory could not be sourced. The rest of the patients in the same ICU were negative for SARS-CoV-2 by RT-PCR. Unvaccinated contacts were advised to remain in quarantine for seven days. No more patients or staff developed the disease after 27th October. It was later found that another physiotherapist who gave physiotherapy to the patient on 15th October had also turned positive on the same day but tested at another lab.

## Materials and methods

**Figure 1:**
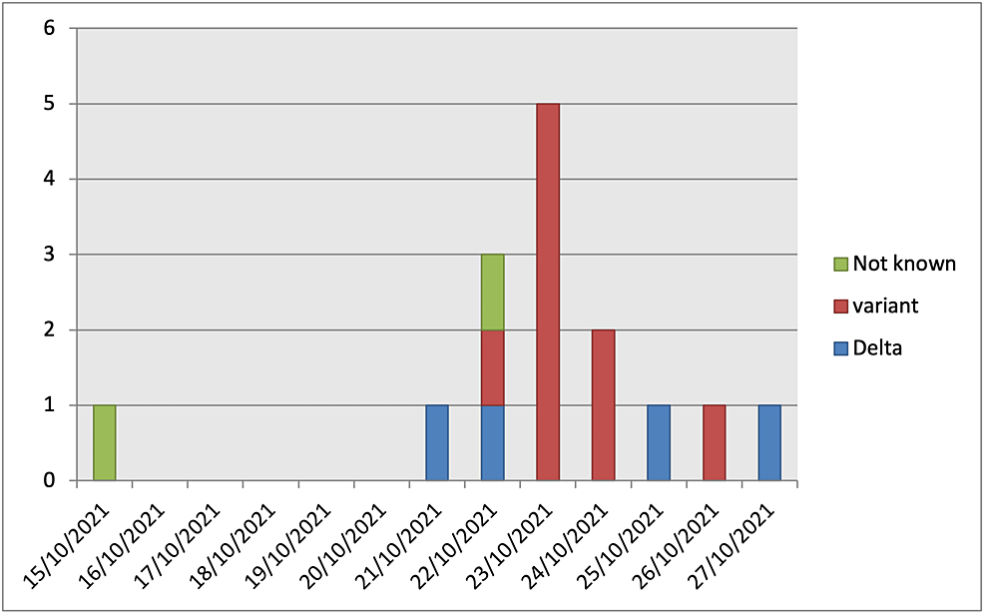
Timeline of cases Timeline of cases shows the number of cases on each day. 'Variant' (red) represents the strains that carried the mutation N501I. The delta strains that did not carry N501I are in blue. Samples from two cases could not be sequenced (green) as they were tested in another laboratory

Epidemiological analysis: data were collected using questionnaires to find the likely source and mode of spread. All those who were positive were included in the data collection. The variables tested were adherence to wearing masks during patient care, proximity to the patient, location and air quality, mode of taking refreshments, and place of stay. A timeline of the development of the cluster was constructed (Figure 1).

Genomic analysis: The nasopharyngeal samples in viral transport media from the CH unit were processed in the COVID-19 testing laboratory for performing RT-PCR for SARS-CoV-2 as per the ICMR guidelines. All available samples from the unit that showed a positive result by RT-PCR with a cycle threshold (Ct) value ≤ 30 (n=13) were further subjected to tSS by capillary electrophoresis. Briefly, viral RNA isolated from throat swab samples was subjected to reverse-transcription PCR (RT-PCR) amplification of the SARS-CoV-2 receptor-binding domain (RBD) region using the AccesQuik RT-PCR system (Promega, Wisconsin, USA) as per the manufacturer’s directions. The primers Cov2SSeq3F 5’ACTTTAGAGTCCAACCAACAGA3’ and Cov2SSeq4R 5’GACTCAGTAAGAACACCTGTGC3’ that amplify a 714 bp region (amino acids 324-547) of the spike protein were used for amplification. The PCR products were purified using the QiaQuick Gel band purification system (Qiagen, Hilden, Germany). The purified products were subjected to Sanger's dideoxy sequencing using BigDye terminator cycle sequencing System v3.1 (Applied Biosystems, Thermo Fisher Scientific, CA, USA) and were analyzed in an ABI310 genetic analyzer (Applied Biosystems, Thermo Fisher Scientific, CA, USA). High-quality reads were compared with the sequences of the SARS-CoV-2 reference strain (GenBank Accession No. NC_045512). Multiple sequence alignments of the translated amino acid sequences of the RBD regions were performed using the BioEdit Suite v.7.2. (https://bioedit.software.informer.com), and variants were identified. The amino acid sequences of the outbreak isolates were compared with the Indian strains deposited in the GenBank. The FASTA sequences of the Indian strains deposited in GenBank from January 2021 to December 2021 were retrieved using a simple GenBank search. The sequences were aligned with the study sequences using BioEdit. Further, the phylogenetic tree was created using the maximum likelihood method to understand the relationship of the outbreak strains to the existing strains retrieved from GenBank using MEGA V.11.0.13 [2].

## Results

An epidemiological investigation showed that eleven subjects out of 72 tested with a history of proximity had disease, while only two with the disease did not have close proximity. Other variables were not significant.

**Figure 2:**
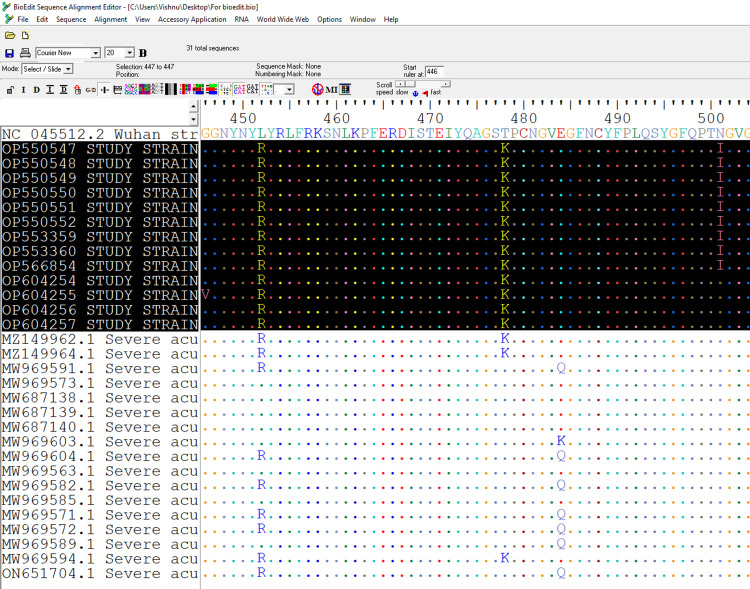
Multisequence alignment of the viral strains The screengrab shows the multisequence alignment of the outbreak strains (highlighted in black) and the other Indian strains with respect to the reference strain (NC045512). Among the 13 outbreak strains, nine strains carried a mutation at the 501st amino acid position of the spike RBD, where asparagine (N) was changed to isoleucine (I), N501I

Sequencing data revealed all viral strains to be Delta variants with characteristic L452R and T478K mutations. However, in nine of the 13 strains (patients and eight HCWs), the RBD carried a unique mutation, N501I (GenBank accession numbers OP550547-52, OP553359-60, and OP566854). Here, the asparagine in the wild type was replaced by an isoleucine in the 501st position. This was not found in the other four strains (GenBank accession OP604254-57) belonging to the mother and three HCWs, namely, CG, N6, N7, and N2 (Figure 2).

**Figure 3:**
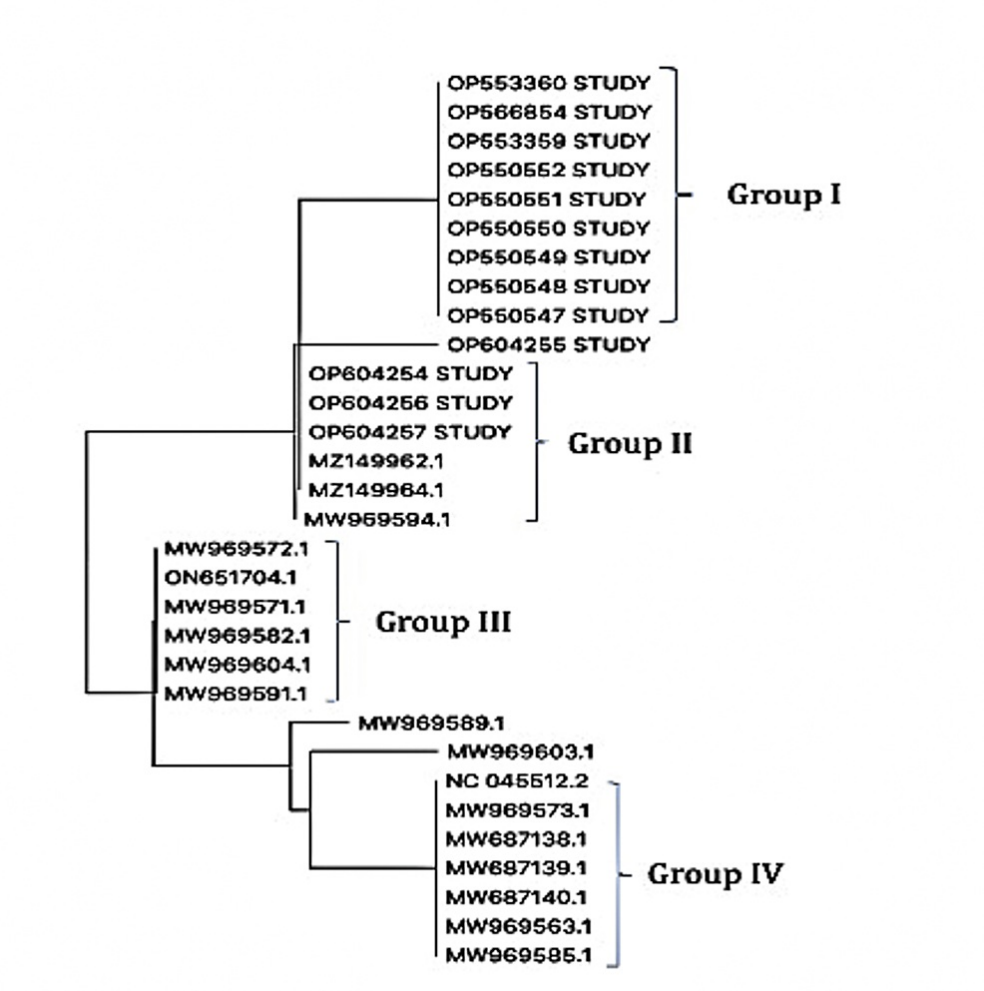
Phylogenetic tree The maximum likelihood phylogenetic tree of the sequences shows all the sequences analyzed in the study. Group I constituted all the 501I outbreak strains. The three other study strains that did not carry the N501I mutation clustered separately.

The amino acid sequences of the outbreak isolates were compared with the Indian strains deposited in GenBank. The FASTA sequences of the Indian strains deposited in GenBank from January 2021 to December 2021 were retrieved using a simple GenBank search. The sequences were aligned with the study sequences using BioEdit. Furthermore, a phylogenetic tree was created using the maximum likelihood method to understand the relationship of the outbreak strains to the existing strains retrieved from GenBank using MEGA V.11.0.13 [2]. Sequence alignment with other Indian strains from GenBank revealed different variants, including Delta, with characteristic L452R/T478K/E484Q mutations. However, a strain carrying N501I could not be identified in GenBank during the period of this cluster. The phylogenetic tree showed the clustering of all the study strains with the N501I mutation (Group I). The non-N501I Delta strains carrying both L452R and T478K mutations clustered with each other (Group II). Similarly, strains with L452R and E484Q mutations clustered together (Group III), while those with only either of these mutations were separated from the others. One of the delta strains from the study that carried the G446V mutation was also separately aligned. The non-delta Indian strains clustered with the NC_045512 reference strains, indicating identity with the wild type (Group IV). The tree with a scale length of 0.01 is shown in Figure 3.

## Discussion

In 1977, Sanger published his landmark paper on a novel method of sequencing the genome. He noted that 15-200 nucleotides can be determined with reasonable accuracy, and if the gels are read further, up to 300 nucleotides from the priming site can be determined [3]. With the advent of the COVID pandemic, next generation sequencing (NGS) gained prominence; however, the existing databases show a lot of gaps since data from developing countries is very scarce due to the considerable cost and skill demand entailed in NGS. This has prompted the use of Sanger sequencing, the gold standard in genome sequencing, for analyses of emerging virus variants [4]. An article by Salles TS et al. highlights the advantage of Sanger sequencing in monitoring S-gene mutations. They also conclude that monitoring the S-gene mutations is crucial for control measures and future vaccine evolution [5]. In Japan during the pandemic, local outbreaks were frequent in 2021, and a new form of ORF8 deletion in Hiroshima provided crucial information on the viral factor involved in local outbreaks [6]. Hence, utilization of tSS in the RBD region was considered for molecular epidemiological characterization when a small cluster of COVID-19 cases developed in our non-COVID-19 superspecialty hospital. The outbreak was unusual since all healthcare workers were vaccinated, and the location was an intensive care unit for post-operative patients where there was a high degree of adherence to infection control precautions. To contain the cluster, epidemiological analysis supported by tSS was envisaged.

The mother of a post-operative patient admitted to the pediatric cardiac surgery intensive care unit tested positive for SARS-CoV-2 by RT-PCR, setting off the screening of the entire unit (n=72). The patient (her son) and 13 staff became positive over a time period of two weeks (15th to 27th October 2021). Two of them were nurses who tested positive on the same day as the patient, and one was a physiotherapist who tested positive five days before. The patient had been in the hospital since March 2021 and could only have acquired the infection from either an external source (like the mother) or from an HCW. A tSS of the available samples (13) was done, and its data analysis showed that all positive cases were due to the Delta variant. However, differences in the mutation pattern were observed between the strains. While all strains had two of the characteristic Delta variant mutations, i.e., L452R and T478K, nine strains had an additional mutation, namely N501I (Figure 2). This shows that not all the positive cases were part of the same cluster. This led to the possibility of ruling out the mother as part of the cluster; however, mutation of the virus in the child after spread is another probable reason that was deemed to be very unlikely. Of the two nurses who became positive on the same day, one was tested and did not carry the mutation. The sample from the other nurse was not available for testing. Hence, the cluster consisted only of the patient and eight nurses with the same mutation in the virus. The study data did not show any significant difference in morbidity between the strains that carried N501I when compared to the other delta strains. Additionally, parameters like age and gender did not carry any significance since this was a study that involved a specific setting. The study data did not show any significant difference in morbidity between the strains that carried N501I when compared to the other delta strains. Additionally, parameters like age and gender did not carry any significance since this was a study that involved a specific setting that included only HCWs and patients as the participants. 

The N501I mutation in SARS-CoV-2 was first reported in the peer-reviewed literature by Sallam et al. (2021) in a single sequence from the first Jordan lineage, B.1.1.312 [7]. It is established that the viral spike protein interacts with the host angiotensin-converting enzyme 2 (ACE2) receptors, establishing a link that is essential for the viral transmission and pathogenesis of COVID-19. The hotspot residue 501 is an important member of the group of strongly attracting amino acid pairs at this protein-protein interaction (PPI) interface and, therefore, is continuously undergoing changes in order to increase virulence and/or transmissibility due to positive selection pressure [8]. The naturally occurring variants with change at residue position N501 are N501Y, N501S, N501I, and N501T, and show neutral or stabilizing effects on the RBD structure. Yadav et al. and the ICMR team in 2021 had retrieved four sequences from travelers from Africa in March 2021 that carried the N501Y mutation in the spike gene [9]. The S protein with these RBD variants increases RBD stability and ACE2 binding affinity but has no effect on its function, as predicted by Verma et al. (2021) using in-silico studies [10]. The more common N501Y mutation, where asparagine is changed to tyrosine, is present in one or more of the World Health Organization (WHO) variants of concern (VOC): Alpha (B.1.1.7), Beta (B.1.351), Gamma (P.1), and Delta (B.1.617.2). The N501Y mutation is known to increase the host ACE2 receptor affinity for the virus and has been shown to accelerate virus replication in human upper-airway cells, thus increasing transmission and virulence. This mutation is said to result in the highest binding affinity between RBD and ACE2 [11], and has been demonstrated to lead to increased morbidity in aged and obese mice [12]. However, the amino acid change from asparagine to isoleucine (N501I), found in this study, has the highest stabilizing effect on the RBD [10]. It also has increased binding affinity towards the host receptor, like the other N501 variants [8]. The mutation detected by tSS in this study was common only to the patient and HCWs who worked in proximity, showing a possible breach of infection control practices. The limitation of this study is that only one outbreak (13 samples) has been studied. In the outbreak, two samples could not be sourced. Whole genome sequencing of the samples was not attempted since it would involve more time and expertise that was not easily accessed in a pandemic situation. Outbreaks occur sporadically and cannot be predicted in most cases. Therefore, it is necessary to develop simpler methods that can be employed on an emergency basis, to define mutations that might throw light on the mode of spread, and to differentiate cases that belong in the cluster versus the ones that are part of the pandemic [13]. In a study reported in 2022, Ko et al. demonstrated the reliability and effectiveness of Sanger sequencing to screen many samples in an outbreak-like situation to look for notable variants of the virus. They found Sanger-based approaches to be practically more suited for the identification of emerging variants since they are more feasible and universally applicable than NGS, especially in countries and locations where they are not affordable. Their later study also pointed out that the genomic information coverage among confirmed COVID-19 cases is too low when using NGS. It reiterated the usefulness of Sanger sequencing not just in understanding variant distribution over time but also in providing evidence for policy making and the formulation or modification of preventive strategies [14,15]. In this study, a similar Sanger-based approach was adapted to a much smaller setting, like our non-COVID hospital.

## Conclusions

In this study, the authors would like to place on record that in this outbreak, tSS provided a rapid and cost-effective solution to correctly delineate between the actual cases that form the cluster and other community cases in a pandemic situation. This finding helped to show that the outbreak was actually nosocomial in origin and to give instructions to strengthen infection control practices like stricter N95 mask compliance and handwashing, which then led to the rapid containment of the outbreak. However, more studies are needed to further authenticate tSS as a technique to clearly demarcate the cases in small clusters that develop in closed communities, like a healthcare facility, within a pandemic.

## References

[1] Mehandru S, Merad M (2022). Pathological sequelae of long-haul COVID. Nat Immunol.

[2] Kumar S, Stecher G, Li M, Knyaz C, Tamura K (2018). MEGA X: Molecular evolutionary genetics analysis across computing platforms. Mol Biol Evol.

[3] Sanger F, Nicklen S, Coulson AR (1977). DNA sequencing with chain-terminating inhibitors. Proc Natl Acad Sci U S A.

[4] Alhamlan FS, Bakheet DM, Bohol MF (2023). SARS-CoV-2 spike gene Sanger sequencing methodology to identify variants of concern. Biotechniques.

[5] Salles TS, Cavalcanti AC, da Costa FB (2022). Genomic surveillance of SARS-CoV-2 Spike gene by sanger sequencing. PLoS One.

[6] Ko K, Nagashima S, E B (2021). Molecular characterization and the mutation pattern of SARS-CoV-2 during first and second wave outbreaks in Hiroshima, Japan. PLoS One.

[7] Sallam M, Mahafzah A (2021). Molecular analysis of SARS-CoV-2 genetic lineages in Jordan: Tracking the introduction and spread of COVID-19 UK variant of concern at a country level. Pathogens.

[8] Khan A, Hussain S, Ahmad S (2022). Computational modelling of potentially emerging SARS-CoV-2 spike protein RBDs mutations with higher binding affinity towards ACE2: A structural modelling study. Comput Biol Med.

[9] Yadav PD, Gupta N, Nyayanit DA (2021). Imported SARS-CoV-2 V501Y.V2 variant (B.1.351) detected in travelers from South Africa and Tanzania to India. Travel Med Infect Dis.

[10] Verma J, Subbarao N (2021). Insilico study on the effect of SARS-CoV-2 RBD hotspot mutants' interaction with ACE2 to understand the binding affinity and stability. Virology.

[11] Tao K, Tzou PL, Nouhin J (2021). The biological and clinical significance of emerging SARS-CoV-2 variants. Nat Rev Genet.

[12] Rathnasinghe R, Jangra S, Cupic A (2021). The N501Y mutation in SARS-CoV-2 spike leads to morbidity in obese and aged mice and is neutralized by convalescent and post-vaccination human sera. medRxiv.

[13] Lim HJ, Park MY, Jung HS (2021). Development of an efficient Sanger sequencing-based assay for detecting SARS-CoV-2 spike mutations. PLoS One.

[14] Ko K, Takahashi K, Nagashima S (2022). Mass screening of SARS-CoV-2 variants using Sanger sequencing strategy in Hiroshima, Japan. Sci Rep.

[15] Ko K, Takahashi K, Nagashima S (2022). Exercising the Sanger sequencing strategy for variants screening and full-length genome of SARS-CoV-2 virus during alpha, delta, and omicron outbreaks in Hiroshima. Viruses.

